# Toward a Biological, Psychological and Familial Approach of Eating Disorders at Onset: Case-Control ANOBAS Study

**DOI:** 10.3389/fpsyg.2021.714414

**Published:** 2021-09-09

**Authors:** Ana Rosa Sepúlveda, Alba Moreno-Encinas, José Angel Martínez-Huertas, Dimitra Anastasiadou, Esther Nova, Ascensión Marcos, Sonia Gómez-Martínez, José Ramón Villa-Asensi, Encarna Mollejo, Montserrat Graell

**Affiliations:** ^1^Department of Biological and Health Psychology, School of Psychology, Autonomous University of Madrid, Madrid, Spain; ^2^Department of Cognitive Psychology, School of Psychology, Autonomous University of Madrid, Madrid, Spain; ^3^Immunonutrition Research Group, Department of Metabolism and Nutrition, Institute of Food Science, Technology and Nutrition (ICTAN), Spanish National Research Council (CSIC), Madrid, Spain; ^4^Pediatric Pneumology, Niño Jesús University Children’s Hospital, Madrid, Spain; ^5^Psychiatry Service, Hospital Universitario del Sureste, Arganda del Rey, Spain; ^6^Department of Child and Adolescent Psychiatry and Psychology, Niño Jesús University Children’s Hospital, Madrid, Spain; ^7^CIBERSAM, Madrid, Spain

**Keywords:** eating disorders, case-control study, biological correlates, psychological correlates, familial correlates

## Abstract

Eating disorders (ED) are considered as heterogeneous disorders with a complex multifactor etiology that involves biological and environmental interaction.

**Objective:** The aim was to identify specific ED bio-psychological-familial correlates at illness onset.

**Methods:** A case-control (1:1) design was applied, which studied 50 adolescents diagnosed with ED at onset (12–17 years old) and their families, paired by age and parents’ socio-educational level with three control samples (40 with an affective disorder, 40 with asthma, and 50 with no pathology) and their respective families. Biological, psychological, and familial correlates were assessed using interviews, standardized questionnaires, and a blood test.

**Results:** After performing conditional logistic regression models for each type of variable, those correlates that showed to be specific for ED were included in a global exploratory model (*R*^2^ = 0.44). The specific correlates identified associated to the onset of an ED were triiodothyronine (T3) as the main specific biological correlate; patients’ drive for thinness, perfectionism and anxiety as the main psychological correlates; and fathers’ emotional over-involvement and depression, and mothers’ anxiety as the main familial correlates.

**Conclusion:** To our knowledge, this is the first study to use three specific control groups assessed through standardized interviews, and to collect a wide variety of data at the illness onset. This study design has allowed to explore which correlates, among those measured, were specific to EDs; finding that perfectionism and family emotional over-involvement, as well as the T3 hormone were relevant to discern ED cases at the illness onset from other adolescents with or without a concurrent pathology.

## Introduction

Eating disorders (ED) are severe psychiatric disorders characterized by pathological attitudes and behaviors related to food. All of them share a common major characteristic: the over-evaluation of shape and weight, and their control. Other common traits are body dissatisfaction and a persistent desire for thinness, which are present throughout the course of the illness [American Psychiatric Association (APA), 2013]. EDs usually begin in early adolescence, being the most frequent diagnosis among adolescents in mental health inpatient units and the third most common chronic illness in female adolescents (Nicholls et al., 2011). Although previous studies have expanded our knowledge about risk factors associated with ED, few have been able to answer whether those risk factors were general or specific to ED psychopathology (Fairburn et al., 1999; Pike et al., 2008; Machado et al., 2014; Gonçalves et al., 2016).

Regarding biological variables, pubertal status, excess body fat mass, and fluctuations in weight are factors associated with ED (Bakalar et al., 2015). Changes in biological variables have been broadly related to homeostatic adaptations to malnutrition, although previous studies have also proposed that some of these, such as appetite-regulating hormones, also contribute to the development and maintenance of different behaviors related to ED (Monteleone and Maj, 2013; Misra and Klibanski, 2014). Peripheral signals, such as fat mass derived hormones and gastrointestinal peptides may act on the central nervous system to influence eating behaviors, energy balance, and mood. In addition, the interactions between leptin, cortisol, and cytokine levels appear to be important mediators in an ED onset and its course, but their true relevance as primary or secondary alterations is mostly unknown (Elegido et al., 2017).

Regarding psychological variables, multiple studies have identified perfectionism as one of the most relevant risk factors of this population (Culbert et al., 2015). Another well-known risk factor for ED is negative affectivity (Dakanalis et al., 2017) which has been shown to persist even after recovery (Klump et al., 2000). However, whereas Fairburn et al. (1999) and Stice (2002) identified perfectionism and negative affectivity as specific risk factors for ED, another study considered perfectionism as a correlate and negative affectivity as a non-specific risk factor (Jacobi et al., 2004). Body dissatisfaction was also found to be an important predictor of ED (Stice et al., 2011; Jacobi and Fittig, 2012). Related to it, shape and weight concern has been confirmed as one of the most potent factors for the onset of an ED (Keel and Forney, 2013).

On the other hand, different familial factors have been identified, such as familial pressure and discord (Fairburn et al., 1999; Pike et al., 2008), teasing (Neumark-Sztainer et al., 2007), negative perception of parental attitudes (Kluck, 2010; Parks et al., 2017), high expressed emotion, and family history of ED (Sepúlveda et al., 2012, 2014; Hilbert et al., 2014). Furthermore, other authors (Le Grange et al., 2010; Machado et al., 2014) have found that familial factors, except family history of ED, were non-specific factors as they were related to increased risk of general psychopathology. Moreover, one of the inherent difficulties of research on familial risk factors of ED is the overrepresentation of mothers’ data, as analyzing the contribution of each parent separately could improve the knowledge about the whole family system (Anastasiadou et al., 2014; Gonçalves et al., 2016).

Previous ED etiological models have agreed that the etiology is complex, and includes biological, psychological, and socioenvironmental factors interacting at the onset and maintenance of the ED (Treasure et al., 2008, 2020). The aim of this article was to identify specific biological, psychological, and familial ED correlates associated with the onset of the disorder. Following the Kraemer et al. (1997)’s risk factors classification, correlates are the kind of factors that cannot demonstrate precedence over the outcome. To evaluate the specificity of these correlates, three control samples were chosen: affective disorders (AD group), asthma pathology (AP group), and a non-pathological group (NP group). EDs present high comorbidity with affective disorders (Ferreiro et al., 2011), suggesting that common and specific ED factors could be pointed out. Asthma sufferers present similarities on the familial level, as both disorders are considered chronic psychosomatic diseases, present severe attacks, which can be life-threatening, and pose high demands of care representing a significant impact on the physical and psychological wellbeing of the families (Theodoratou-Bekou et al., 2012; Verkleij et al., 2015). A control group without pathology was selected in order to control the role of the adolescence as an important risk factor for an ED (Keel and Forney, 2013). Consistent with the scientific literature, we hypothesized that some biological correlates (biochemical, neuroendocrine, and immunological), some psychological correlates (attitudes and behaviors related to eating psychopathology, body dissatisfaction, perfectionism and anxious, depressive, and obsessive symptomatology), and some familial correlates (family functioning, expressed emotion and anxious, depressive, and obsessive symptomatology) were specific correlates associated with the onset of an ED. We also hypothesized that an exploratory bio-psycho-familiar model based on these specific correlates would allow to identify the ED group.

## Materials and Methods

### Design and Procedure

The current research follows a cross-sectional case-control design, using an ED group as the case group and matching with three control groups by sex and age of the patients and socioeconomic status of the parents, following the Hollingshead Redlich Scale (Hollingshead and Redlich, 1953). A complete sample description of the ANOBAS protocol and a detailed explanation of the suitability of the study control groups is provided in Sepulveda et al. (2021).

The recruitment was carried out during 4 years. Firstly, the ED sample was recruited at the outpatient Eating Disorders Unit at the Niño Jesús University Children’s Hospital in Madrid, Spain. The samples for the three control groups was then recruited, in order to match the characteristics of each ED adolescent (1:1). The AD sample was recruited at different Mental Health Centers in the Community of Madrid. In both psychiatric groups, patients had been diagnosed by mental health professionals. In addition, AP participants were recruited at the Pneumology Department at the Niño Jesús University Children’s Hospital and the NP group was recruited at different schools in the Community of Madrid. Short telephone interviews were conducted to confirm the sociodemographic variables, and once informed written consent was obtained from adolescents and their parents, the cases were matched. The first assessment included a socio-demographic interview, a semi-structured psychiatric interview to confirm the previous diagnoses and to assess new possible comorbid psychiatric diagnoses, and a battery of questionnaires for both parents and daughters. Participants had 1 week to complete the questionnaires. This assessment was followed by a full blood test. The blood sample was collected at the Niño Jesús University Children’s Hospital and evaluated by the Immunonutrition Group at the Institute of Food Science, Technology and Nutrition (ICTAN-CSIC). Fasting venous blood samples were collected between 8 and 9 AM from patients and controls in an EDTA-K3E Vacutainer (BD Biosciences) tubes. Plasma was obtained by centrifugation during 15 min at 1,300 *g* and 4°C. Aliquots were frozen at −80°C until analysis.

Confidentiality was guaranteed for all the participants. The study received ethical approval by the Hospital Ethics Committee (Ref. Code, R-0009/10), and the corresponding University Research Ethics Committee (UAM, CEI 25-673).

### Participants

The sample was made up by 180 females, with ages between 12 and 17 years, and their parents. Four groups were recruited: 50 adolescents diagnosed with an ED at onset of the illness, 40 adolescents diagnosed with an affective disorder at onset (AD group), 40 adolescents diagnosed with severe asthma pathology (AP group), and 50 adolescents without a pathology (NP group). Depending on the sample, data was collected from between 30 and 40 fathers, and 40 and 50 mothers.

Exclusion criteria for all groups were to suffer any metabolic conditions known to influence Body Mass Index (BMI) or a psychotic disorder, and for the three control groups, to present an ED or a BMI above 30 or below 17.5. Inclusion criteria for the ED and AD group were to present an early stage of the illness at first diagnosis (a year or less of illness duration). Inclusion criteria for the AD group were to present a diagnosis of an affective disorder without ED diagnosis. Inclusion criteria for the AP group were to have been diagnosed before the age of 7 with asthma and to have visited at least three times an emergency service, which allowed us to select more severe asthma cases. Overall, nine participants were excluded from the study after the assessment because of co-occurrence of ED and AD (*n* = 2), co-occurrence of ED or AP (*n* = 2), presence of psychosis (*n* = 1), presence of a metabolic disorder (*n* = 1), and ED pathology in the NP group (*n* = 3). All of the excluded participants were not considered for matching.

Regarding the sample size, taking into account weight concerns assessed through the Eating Disorders Inventory (Garner, 1991), considered as one of the most well-supported risk factor for ED, a mean effect size of AUC = 0.746 was found in one of the main reviews about risk factors in this pathology (Jacobi et al., 2004). Based on that mean effect size, the Cohen’s *d* was calculated (*d* = 0.936). The G^∗^Power program was used in order to calculate the sample size needed to detect this effect, obtaining an estimated sample size per group of 27. Based on these suggestions, a sample size of 40 or 50 was considered enough to reach good effect sizes.

### Measures

#### Diagnostic Assessment

Current and lifetime psychiatric disorders were evaluated with the Kiddie-Schedule for Affective Disorders and Schizophrenia Interview (K-SADS-PL; Kaufman et al., 1997); a semi-structured interview developed to diagnose children and adolescents using DSM-IV diagnoses. Diagnoses were adapted to the DSM-5 [American Psychiatric Association (APA), 2013].

#### Biological Assessment

A physical examination and laboratory analysis of blood markers related to nutritional and immunological status were assessed, including the following types of variables:

(a) Anthropometric variables: weight, height, and BMI.
(b) Biochemical variables: alkaline phosphatase, total cholesterol, ferritin, vitamin B12, with automated analyzer using colorimetric and nephelometric techniques and by electric potential using a selective electrode (Na, K).
(c) Neuroendocrine and Immunological variables: free tetra-iodothyronine (T4), tri-iodothyronine (T3), cortisol, estradiol, insulin like growth factor-1 (IGF1), IGF-binding protein-3 (IGFBP3), complement component 3 (C3), tumor necrosis factor α (TNF-α), leptin, soluble leptin receptor, adiponectin, by RIA, ELISA, and xMAP Technology for immunoassay of multiple analyses (Millipore).

Blood collected in EDTA-K3 vacumtainers was used for a lymphocyte subset analysis. Immediately after collection, 1 mL of blood was mixed with an equal volume (1 mL) of preservative solution and refrigerated for 2–6 days for processing and flow cytometry analysis. Unfortunately, due to budget limitations, the asthma group did not have their immunological variables measured.

#### Psychological Correlates Assessment

Each adolescent completed a battery of different instruments, which have shown adequate psychometric validity in Spanish adolescent samples (in the current study Cronbach α ranged between 0.81 and 0.98). Attitudes and behaviors related to eating psychopathology were assessed with the Eating Disorders Inventory-II (EDI-II; Garner, 1991). Body dissatisfaction was evaluated with the Body Shape Questionnaire (BSQ; Cooper et al., 1987). Depression was assessed with the Child Depression Inventory (CDI; Kovacs, 1992); anxiety with the State-Trait Anxiety Inventory for Children (STAIC; Spielberger et al., 1973) and obsessiveness with the Leyton Obsessional Inventory-Child Version (LOI-CV; Berg et al., 1986). Lastly, we used the Child Adolescent Perfectionism Scale (CAPS; Flett et al., 2000) to evaluate perfectionism.

#### Familial Correlates Assessment

The parents of each participant completed a battery of five questionnaires. These measures have shown adequate psychometric validity across Spanish populations (in the current study Cronbach α ranged between 0.78 and 0.92). To evaluate the psychological well-being of the parents we used the Beck Depression Inventory (BDI; Beck et al., 1961) to assess depressive symptoms, the State-Trait Anxiety Inventory (STAI, Spielberger et al., 1970) to assess the level of anxiety, and the Obsessive-Compulsive Inventory-Revised (OCI-R; Foa et al., 2002) to assess obsessive-compulsive symptoms. Regarding family functioning variables, we used the Family Adaptability and Cohesion Scale (FACES-II; Olson et al., 1982) to assess adaptability and cohesion, and the Family Questionnaire (FQ; Wiedemann et al., 2002) to evaluate critical comments (CC) and emotional over-involvement (EOI).

### Data Analysis

All the statistical analyses were performed with R software.

Firstly, conditional logistic regressions were conducted for each risk factor using the *survival* package. Conditional logistic regressions compare each ED participant with her matching case-control participant from the AD, AP, and NP groups. More specifically, each ED participant was compared with a strata of her matching case-control AD, AP, and NP participants. Conditional logistic regressions were then conducted for each group of biological, psychological, and familial correlates where a stepwise model selection was applied to select the most relevant correlates in the model using the AIC indices of the *MASS* package. The statistical significance of individual correlates was corrected using Holm–Bonferroni correction for multiple comparisons. All the variables were standardized and no multicollinearity was observed, neither in biological, familial nor psychological models.

Secondly, the same conditional logistic regressions procedure was followed to estimate an exploratory bio-psycho-familial model. In this case, the complexity and the computational burden of the statistical model forced us to impute missing values by the mean of each group (missing patterns were isolated to specific cases, but listwise deletion inherent to conditional logistic regressions would considerably reduce the number of observations). In the bio-psycho-familial model, we only included those correlates that were previously conserved by stepwise model selections using the AIC indices. All the variables were standardized to estimate the bio-psycho-familial model because they had different score ranges. Finally, a stepwise model selection was applied in this model in order to determine the most relevant correlates to discriminate between ED and the control groups (AD, AP, and NP participants).

## Results

### Sociodemographic Characteristics of the Participants

Participants’ sociodemographics are described in Table 1, in which each control sample is compared with the ED sample. Given the design, no differences were found for participants’ age and socioeconomic status of the parents. In addition, no differences were found between the psychiatric groups (ED and AD) for illness duration. We only found statistically significant differences between the groups controlling by their case-control matching in the patients’ BMI. ED participants presented the following diagnoses: anorexia nervosa (AN) restrictive subtype (70%); AN purgative subtype (16%) and other specified feeding and eating disorder (14%). AD participants presented the following diagnoses: major depressive disorder (90%); dysthymia (7.5%); adjustment disorder with depressive symptoms (2.5%).

**TABLE 1:** Descriptive analyses of the sociodemographic characteristics of the participants, and mixed-effects results to test the differences between case-control with individual matching.

	ED (*N* = 50)	AD (*N* = 40)	AP (*N* = 40)	NP (*N* = 50)	Differences between groups
	M (SD)/%	M (SD)/%	M (SD)/%	M (SD)/%	β (*SE*)
**Adolescents**					
Age (years)	14.68 (1.39)	15.10 (1.55)	14.73 (1.74)	14.66 (1.32)	0.00 (0.03)
Body mass index	16.07 (1.74)	22.41 (2.95)	21.24 (2.87)	21.18 (2.66)	6.89 (2.77)**
Length of illness (months)	10.32 (7.41)	11.70 (6.02)	–	–	1.14 (1.28)
Diagnosis	AN-R: 70% AN-P: 16% FEDNEC: 14%	MDD: 90% Dysthymia: 7.5% Adjustment-disorder: 2.5%			–
History of previous psychiatric disorder	14.00%	52.50%	25.00%	8.00%	−0.04 (0.03)
**Parents**					
Father’s age (years)	47.54 (4.79)	48.32 (4.89)	46.67 (4.50)	51.02 (4.44)	0.95 (0.30)**
Mother’s age (years)	45.55 (3.62)	45.73 (4.83)	45.55 (4.15)	48.30 (3.54)	0.84 (0.25)**
Father’s psychiatric antecedent	14.00%	20.00%	10.00%	09.00%	0.02 (0.02)
Mother’s psychiatric antecedent	36.00%	50.00%	28.00%	26.00%	−0.05 (0.03)
Socioeconomic status					0.05 (0.02)
Low	20.00%	27.50%	20.00%	14.00%	
Middle	12.00%	25.00%	20.00%	14.00%	
High	68.00%	47.50%	60.00%	72.00%	

*ED, eating disorders; AD, affective disorder; AP, asthma pathology; NP, non-pathology group; N, sample size; M, mean; SD, standard deviation. AN-R, anorexia nervosa-restricting type; AN-P, anorexia nervosa-purging type; FED-NEC, feeding or eating disorder not elsewhere classified; MDD, major depressive disorder. Statistical differences between groups were estimated through mixed effects models. β, fixed effect; *SE*, standard error.*

**p < 0.01.

### Examining Biological Correlates for Eating Disorders

Conditional logistic regressions were computed for each biological correlate (see Table 2). Results showed that higher values of vitamin B12, IGFBP3, total cholesterol, and adiponectin were relevant to differentiate the ED group with the control groups. On the other hand, the reduced values of T3 and IGF-1 were also relevant to differentiate the ED group with the control groups.

**TABLE 2:** Descriptive analyses and conditional logistic regressions for biological correlates.

	Descriptive analyses				Conditional logistic regression					
	ED (*N* = 50)	AD (*N* = 40)	AP (*N* = 40)	NP (*N* = 50)	Individual correlate			Stepwise model selection		
	M (SD)	M (SD)	M (SD)	M (SD)	OR	95% IC	*z*	OR	95% IC	*z*
Alkaline phosphatase (IU/L)	65.30 (34.81)	71.03 (23.80)	93.53 (52.05)	90.87 (52.96)	0.40	0.20–0.79	−2.63^a^	–	–	–
T3 (ng/mL)	1.00 (0.20)	1.23 (0.17)	1.28 (0.16)	1.26 (0.17)	0.55	0.39–0.78	−3.32**	0.32	0.19–1.00	−2.68**
Free T4 (ng/dL)	0.82 (0.09)	0.87 (0.07)	0.92 (0.08)	0.91 (0.09)	0.74	0.54–1.01	−1.88^a^	0.43	0.14–0.74	−1.96*
Cortisol (μg/dL)	16.86 (5.67)	11.04 (4.15)	9.11 (3.92)	14.46 (4.75)	1.09	0.79–1.52	0.53	–	–	–
Estradiol (pg/mL)	24.65 (39.12)	98.69 (87.38)	91.70 (76.25)	80.61 (65.83)	1.41	1.00–1.98	1.97^a^	–	–	–
C3 (mg/dL)	83.13 (16.43)	100.77 (18.38)	94.10 (13.85)	97.74 (16.15)	1.05	0.76–1.45	0.32	–	–	–
Ferritin (ng/mL)	77.42 (49.26)	28.50 (19.96)	36.27 (22.67)	28.31 (21.87)	1.53	1.08–2.16	2.40^a^	–	–	–
Vitamin B12 (pg/mL)	525.50 (189.91)	408.11 (177.44)	399.73 (157.41)	423.32 (134.84)	1.94	1.29–2.91	3.17**	–	–	–
IGF-1 (ng/mL)	269.32 (153.41)	352.59 (99.54)	387.83 (116.78)	375.22 (88.76)	0.37	0.22–0.60	−3.95**	0.40	0.16–0.98	−2.00*
IGFBP3 (μg/mL)	5.06 (1.02)	5.00 (0.78)	5.00 (0.72)	4.83 (0.70)	1.59	1.13–2.25	2.66**	4.94	1.75–13.95	3.02**
Total cholesterol (mg/dL)	179.46 (41.79)	148.14 (24.64)	146.42 (28.83)	154.85 (26.75)	2.50	1.55–4.02	3.76**	2.98	1.28–6.93	2.54*
Leptin (pg/mL)	1930.30 (2470.39)	6394.81 (3693.47)	–	7498.77 (4903.02)	0.81	0.56–1.19	–1.06			
Soluble leptin receptor (ng/mL)	3.08 (0.44)	3.74 (0.24)	–	3.79 (0.28)	0.65	0.46–0.92	−2.44^a^			
Adiponectin (μg/mL)	35.37 (15.82)	23.86 (18.03)	–	20.33 (7.77)	3.24	2.09–5.02	5.26**			
TNF-α (pg/mL)	3.42 (3.03)	3.34 (0.77)	–	4.42 (1.80)	1.19	0.89–1.60	1.17			

*ED, eating disorders; AD, affective disorder; AP, asthma pathology; NP, non-pathology group; N, sample size; M, mean; SD, standard deviation; OR, odds ratio; Individual correlate, conditional logistic regression for each variable; Stepwise model selection, selected correlates for the model using AIC indices (empty cells mean that the variable was excluded in the model; gray cells mean that the variable was not included in the initial model).*

*^*a*^*z*-value is statistically significant, but its statistical significance was corrected for multiple comparisons.*

*p < 0.05;

**p < 0.01.

*Statistical significance of individual correlates was corrected using Holm–Bonferroni correction for multiple comparisons. Some biological variables were not recollected for the AP group.*

A conditional logistic regression was estimated using all of the biological correlates (except leptin, soluble leptin receptor, adiponectin, and TNF-α because they were not collected in the AP group) as covariates. Stepwise model selection showed that the most relevant variables to differentiate the ED group with the control groups were T3, free T4, IGF-1, IGFBP3, and total cholesterol.

### Examining Psychological Correlates for Eating Disorders

Conditional logistic regressions were computed for each psychological correlate (see Table 3). Results showed that the ED group reported more drive for thinness, body dissatisfaction (BSQ) and self-directed perfectionism than control groups.

**TABLE 3:** Descriptive analyses and conditional logistic regressions for psychological correlates.

	**Descriptive analyses**				**Conditional logistic regression**					
	**ED (*N* = 50)**	**AD (*N* = 40)**	**AP (*N* = 40)**	**NP (*N* = 50)**	**Individual correlate**			**Stepwise model selection**		
	**M (SD)**	**M (SD)**	**M (SD)**	**M (SD)**	**OR**	**95% IC**	***z***	**OR**	**95% IC**	***z***
Drive for thinness (EDI-II)	8.29 (6.92)	6.70 (6.79)	2.48 (3.66)	1.83 (4.13)	2.07	1.42–3.02	3.80**	16.17	2.78–94.06	3.10**
Bulimia (EDI-II)	0.93 (1.80)	2.05 (3.80)	0.63 (1.23)	0.48 (1.24)	0.96	0.68–1.36	–0.23	–	–	–
Body dissatisfaction (EDI-II)	9.39 (6.75)	11.05 (8.36)	5.75 (6.30)	3.81 (5.51)	1.37	1.00–1.87	1.97^a^	–	–	–
Ineffectiveness (EDI-II)	5.62 (5.89)	12.46 (8.27)	2.23 (3.84)	1.63 (3.56)	1.09	0.80–1.49	0.53	0.42	0.14–1.31	–1.50
Perfectionism (EDI-II)	4.06 (3.70)	6.35 (4.64)	4.03 (3.29)	3.50 (3.36)	0.86	0.60–1.24	–0.81	–	–	–
Interpersonal distrust (EDI-II)	4.20 (3.90)	7.43 (4.64)	1.68 (1.61)	2.21 (2.93)	1.19	0.86–1.64	1.06	–	–	–
Interoceptive awareness (EDI)	6.27 (5.72)	11.14 (6.52)	3.20 (4.24)	2.23 (4.18)	1.22	0.88–1.68	1.20	–	–	–
Maturity fears (EDI-II)	8.10 (4.40)	9.27 (5.63)	6.78 (3.69)	7.50 (4.77)	1.13	0.82–1.57	0.73	–	–	–
Body dissatisfaction (BSQ)	94.49 (45.16)	101.75 (47.52)	59.08 (24.79)	62.28 (27.04)	1.56	1.13–2.15	2.67**	0.14	0.03–0.69	−2.43*
Depression (CDI)	14.36 (8.98)	23.48 (8.51)	7.90 (4.16)	8.33 (5.18)	1.13	0.85–1.51	0.82	–	–	–
Anxiety-state (STAIC-S)	35.20 (10.64)	39.10 (8.37)	27.48 (5.75)	26.58 (6.54)	1.46	1.06–2.00	2.33^a^	5.07	1.54–16.64	2.67**
Anxiety-trait (STAIC-T)	38.80 (10.28)	45.53 (7.60)	32.20 (6.57)	32.00 (7.49)	1.24	0.91–1.70	1.35	0.18	0.03–0.91	−2.07*
Obsessive symptoms (LOI)	9.10 (4.67)	10.00 (3.30)	6.83 (3.62)	7.40 (3.80)	1.34	0.94–1.90	1.63	2.34	0.90–6.11	1.74^t^
Self-oriented perfectionism (CAPS)	39.11 (10.95)	36.74 (8.31)	34.33 (8.71)	32.96 (7.81)	1.64	1.15–2.34	2.71**	5.03	1.72–14.69	2.95**
Socially prescribed perfectionism (CAPS)	21.60 (8.97)	28.82 (8.38)	23.62 (7.82)	24.29 (7.83)	0.64	0.44–0.93	−2.35^a^	0.26	0.13–0.55	−3.59**

*ED, eating disorders; AD, affective disorder; AP, asthma pathology; NP, non-pathology group; N, sample size; M, mean; SD, standard deviation; OR, odds ratio; Individual correlate, conditional logistic regression for each variable. Stepwise model selection, selected correlates for the model using AIC indices (empty cells mean that the variable was excluded in the model).*

*^*a*^*z*-value is statistically significant, but its statistical significance was corrected for multiple comparisons.*

*^*t*^*p* < 0.10.*

*p < 0.05;

**p < 0.01.

*Statistical significance of individual correlates was corrected using Holm–Bonferroni correction for multiple comparisons.*

Also, a conditional logistic regression was estimated using all of the psychological correlates as covariates. A stepwise model selection showed that patients with ED reported more drive for thinness, anxiety-state, obsessive symptoms, and self-oriented perfectionism. However, patients with ED reported less body dissatisfaction, trait-anxiety, and socially prescribed perfectionism than the control groups.

### Examining Familial Correlates for Eating Disorders

Conditional logistic regressions were computed for each familial correlate (see Table 4). Results showed that the ED group was more exposed to both fathers’ and mothers’ emotional over-involvement and mothers’ anxiety-state compared to the control groups.

**TABLE 4:** Descriptive analyses and conditional logistic regressions for family correlates.

	**Descriptive analyses**				**Conditional logistic regression**					
	**ED (*N* = 50)**	**AD (*N* = 40)**	**AP (*N* = 40)**	**NP (*N* = 50)**	**Individual correlate**			**Stepwise model selection**		
	**M (SD)**	**M (SD)**	**M (SD)**	**M (SD)**	**OR**	**95% IC**	***z***	**OR**	**95% IC**	***z***
**Father**										
EE: criticism (FQ-CC)	19.89 (3.77)	22.41 (4.96)	19.70 (5.28)	18.03 (4.53)	1.09	0.70–1.70	0.40	0.39	0.17–0.89	−2.25*
EE: emotional over-involvement (FQ-EOI)	26.66 (3.83)	26.30 (5.26)	19.91 (4.70)	18.51 (3.77)	3.29	1.82–5.92	3.96**	7.94	2.72–23.19	3.79**
Cohesion (FACES)	66.75 (9.70)	58.67 (7.70)	68.04 (6.66)	64.33 (7.48)	1.28	0.88–1.87	1.28	–	–	–
Adaptability (FACES)	52.95 (5.78)	50.26 (6.54)	52.04 (6.53)	51.26 (6.26)	1.26	0.86–1.86	1.19	–	–	–
Depression (BDI)	5.34 (4.21)	10.89 (11.04)	4.68 (4.13)	3.59 (2.85)	0.96	0.66–1.40	–0.20	0.23	0.06–0.80	−2.32*
Anxiety-state (STAI-S)	21.16 (9.07)	24.0 (13.21)	14.32 (9.75)	13.28 (6.66)	1.54	1.08–2.21	2.36^a^	2.12	0.84–5.34	1.59
Anxiety-trait (STAI-T)	14.56 (5.96)	20.04 (12.19)	15.16 (7.60)	11.41 (6.25)	1.02	0.72–1.47	0.13	–	–	–
Obsessive symptoms (OCI-R)	11.39 (8.24)	15.19 (12.03)	12.20 (9.65)	11.56 (6.68)	0.94	0.63–1.42	–0.28	–	–	–
**Mother**										
EE: criticism (FQ-CC)	18.73 (4.26)	22.73 (5.87)	20.11 (5.25)	18.35 (4.58)	0.78	0.55–1.11	–1.36	0.20	0.07–0.58	−2.93**
EE: emotional over-involvement (FQ-EOI)	25.92 (3.25)	26.30 (5.71)	21.16 (4.19)	17.79 (3.19)	2.20	1.50–3.22	4.01**	5.52	1.96–15.54	3.24**
Cohesion (FACES)	67.33 (9.57)	63.19 (8.67)	70.11 (6.91)	66.75 (6.91)	1.03	0.74–1.44	0.16	0.57	0.26–1.21	–1.46
Adaptability (FACES)	52.83 (6.21)	51.25 (7.94)	52.24 (6.06)	52.27 (4.83)	1.17	0.83–1.65	0.88	–	–	–
Depression (BDI)	7.92 (3.71)	11.32 (7.34)	7.62 (7.30)	5.29 (4.38)	1.06	0.75–1.50	0.32	0.46	0.18–1.22	–1.56
Anxiety-state (STAI-S)	26.75 (9.017)	26.43 (11.49)	17.51 (8.88)	16.40 (7.95)	2.00	1.37–2.91	3.62**	6.09	2.12–17.53	3.35**
Anxiety-trait (STAI-T)	17.50 (8.19)	23.57 (10.37)	18.74 (9.78)	17.12 (8.82)	0.79	0.55–1.15	–1.23	0.17	0.05–0.56	−2.92**
Obsessive symptoms (OCI-R)	11.04 (10.87)	15.95 (9.19)	12.77 (8.38)	11.34 (7.86)	0.75	0.51–1.10	–1.47	–	–	–

*ED, eating disorders; AD, affective disorder; AP, asthma pathology; NP, non-pathology group; EE, expressed emotion; N, sample size; M, mean; SD, standard deviation; OR, odds ratio; Individual correlate, conditional logistic regression for each variable; Stepwise model selection, selected correlates for the model using AIC indices (empty cells mean that the variable was excluded in the model).*

*^*a*^*z*-value is statistically significant, but its statistical significance was corrected for multiple comparisons.*

*p < 0.05;

**p < 0.01.

*Statistical significance of individual correlates was corrected using Holm–Bonferroni correction for multiple comparisons.*

A conditional logistic regression was then estimated using all the familial correlates as covariates. A stepwise model selection showed that patients with an ED were more exposed to fathers’ EOI, mothers’ EOI, and mother’s anxiety-state. However, patients with ED were less exposed to fathers’ criticism and mothers’ criticism, fathers’ depression, and mothers’ trait-anxiety than the control groups.

### An Exploratory Bio-Psycho-Familial Model of Specific Correlates for Eating Disorders

Once all the relevant variables were selected in the previous analyses, a bio-psycho-familial model was estimated (see Table 5). The complexity and thus the computational burden of the full model forced us to remove total cholesterol, IGFBP3, and mothers’ state-anxiety from the full model. The most relevant variables of this full model were selected by a stepwise model selection using the AIC indices. This model was composed by biological, psychological and familial correlates that explained a considerable part of the variance of the dependent variable (*R*^2^ = 0.44). In the case of biological correlates, the reduction in T3 was relevant to differentiate between case-control groups. In the case of familial correlates, we found that the ED group was more exposed to fathers’ emotional over-involvement, and less exposed to fathers’ depression and mothers’ trait-anxiety, compared to control groups. In the case of psychological correlates, we found that the ED group reported more drive for thinness and self-oriented perfectionism, and that they reported less trait-anxiety and socially prescribed perfectionism, compared to the control groups.

**TABLE 5:** Conditional logistic regressions to determine a bio-psycho-familial model of correlates for eating disorders.

		**Conditional logistic regression**					
		**Full model**			**Stepwise model selection**		
		**OR**	**95% IC**	***z***	**OR**	**95% IC**	***z***
**Biological correlates**							
	T3	0.84	0.70–1.02	−1.77^t^	0.87	0.78–0.98	−2.40*
	Free T4	0.93	0.78–1.11	−0.77	–	–	–
	IGF-1	0.99	0.98–1.01	−0.91	–	–	–
**Familial correlates**							
	**Father**						
	Criticism	0.77	0.38–1.56	−0.72	–	–	–
	Emotional over-involvement	5.38	0.93–31.22	1.88^t^	2.74	1.24–6.04	2.50*
	Depression	0.34	0.10–1.09	−1.82^t^	0.50	0.25–0.98	−2.02*
	**Mother**						
	Criticism	1.32	0.77–2.27	1.01	–	–	–
	Emotional over-involvement	0.91	0.67–1.24	−0.60	–	–	–
	Trait-anxiety	0.70	0.45–1.07	−1.65^t^	0.82	0.67–0.98	−1.99*
**Psychological correlates**							
	Drive for thinness	1.40	0.95–2.07	1.68^t^	1.38	1.05–1.80	2.35*
	Body dissatisfaction	1.00	0.95–1.05	−0.02	–	–	–
	Trait-anxiety	0.80	0.61–1.05	−1.61	0.81	0.67–0.99	−2.07*
	Self-directed perfectionism	1.30	1.00–1.70	1.95^t^	1.21	1.03–1.43	2.31*
	Socially prescribed perfectionism	0.65	0.42–1.01	−1.91^t^	0.76	0.60–0.96	−2.30*

*OR, odds ratio; Full model, conditional logistic regression with all the variables; Stepwise model selection, selected correlates for the model using AIC indices (“–” means that the variable was excluded in the model).*

*^*t*^*p* < 0.10.*

*p < 0.05.

*Given the complexity of the full model, missing data was imputed with the mean of each group to estimate both models.*

## Discussion

To our knowledge, this is the first study to use three specific control groups, with standardized interviews for all the participants, collecting wide variety of data that included the capture of family functioning from both parents’ perspectives. Furthermore, ED patients were recruited at the onset of their illness, something that helped us to identify specific correlates associated with the development of an ED, in order to generate an exploratory bio-psychological-familial model.

Regarding biological variables, the biochemical variables vitamin B12 and total cholesterol, as well as the neuroendocrine variables T3, IGF1, IGFBP, and adiponectin, were relevant to differentiate the ED group with the control groups. However, when all the biological variables were considered conjointly, all these variables except vitamin B12 and adiponectin, appeared with free T4 to be the most relevant specific correlates associated with the onset of an ED. These are all endocrine variables directly related to energy availability for metabolic functions. T3 and Free T4 are usually low in AN patients in order to decrease energy requirements, while IGF-1 is a hormone produced in many cells in response to the growth hormone, it has widespread metabolic functions and is greatly involved in the adaptation to starvation (Misra and Klibanski, 2014). IGF-1 has been shown to decrease in acute stages of AN, IGFBP-1 is increases, and IGFBP-3 levels are less clear. The high levels of cholesterol has been related to the decrease of T3 and T4 (Matzkin et al., 2007; Himmerich et al., 2019). In addition, a trend toward the normalization of these molecules with anthropometrical recovery has been shown (Støving et al., 2007). Thus, it appears that these molecules are good correlates to identify ED patients with a low BMI who have been recently diagnosed, and are, thus, under the effects of maintained restrictive behaviors. However, their usefulness as a potential predictor is low since their alteration is believed to be secondary to malnutrition.

The psychological correlates that have shown to be specific correlates for ED were drive for thinness, body dissatisfaction, and self-oriented perfectionism. However, when all the psychological variables were considered conjointly, the role of body dissatisfaction was not maintained and other correlates, such as obsessive symptoms, anxiety, and socially prescribed perfectionism, appeared as important correlates. Whereas body dissatisfaction was found as an important predictor for ED (Stice et al., 2011), it could also act as a predictor for an affective disorder (Ferreiro et al., 2011; Bornioli et al., 2021). In addition, their prevalence is high in adolescence (Stice, 2002) and mainly in females (Al Sabbah et al., 2009), and it appears to not be a specific correlate. Furthermore, other researchers’ findings underline the role of perfectionism in ED (Fairburn et al., 1999; Pike et al., 2008; Machado et al., 2014), similar to our findings. Nevertheless, an important difference was found between self-oriented perfectionism and socially prescribed perfectionism. Castro-Fornieles et al. (2007) found that self-oriented perfectionism was more specific for EDs and socially prescribed perfectionism appeared in similar levels in other psychiatric disorders.

On the other hand, emotional over-involvement of both parents and mother state-anxiety emerged as specific familial correlates for an ED. When all the familial variables were considered conjointly, the reduction of both parents’ criticism, fathers’ depression and mothers’ trait-anxiety appeared as specific correlates for ED. These results may suggest that psychological distress (characterized by severe depression and trait-anxiety) and high expressed emotion of family members may be associated with an ED, consistent with the review done by Zabala et al. (2009). Likewise, the difference in the dimensions of expressed emotion is in accordance with the tendency of higher levels of EE-EOI compared to EE-CC in families with an ED, a finding which was reported by Anastasiadou et al. (2016).

The exploratory bio-psychological-familial model showed that the increase of fathers’ EOI, patients’ drive of thinness and self-oriented perfectionism together with the decrease of T3, anxiety and socially prescribed perfectionism of the adolescents as well as the decrease of fathers’ depression and mothers’ anxiety were specifically associated to the onset of an ED. The fathers’ EOI appeared as a robust specific correlate, in contrast to a recent systematic review which proposed that mothers were more emotionally over-involved than fathers, who tend to be more critical (Anastasiadou et al., 2014). However, in this review several studies did not include comparison groups. In our research, mothers of the AD group showed higher levels of emotional over-involvement as well as fathers for the AD group showed higher levels of criticism compared to parents with ED. It seems that in the ED group, fathers play an important role, which can differentiate this group from other control groups even better than mothers, suggesting that future research should include them in the assessment.

These results also contrast with the studies that have suggested that familial factors are non-specific factors for the onset of an ED (Le Grange et al., 2010; Herpertz-Dahlmann et al., 2011; Machado et al., 2014). Indeed, some authors have emphasized the possibility that these factors would be an accommodation to the illness rather than predisposing factors that explain it (Le Grange et al., 2010). Regardless of their role, expressed emotion is a potential prognostic indicator, that is stable in periods of up to 2 years and that predicts poor outcomes for treatment (Peris and Miklowitz, 2015). Further studies are needed in order to clarify the role of familial correlates in ED.

In addition, the decrease of fathers’ levels of depression and mothers’ trait anxiety followed a similar tendency as the adolescents’ decreased in the trait-anxiety. Several studies have examined the similarities between the negative affectivity dimension and the factors measured by BDI or STAI scales, and have concluded that they should be considered as a measure of general negative affect (Balsamo et al., 2013). Therefore, our findings do not support the centrality of negative affectivity as a specific correlate for ED, in concordance with similar recent studies that have considered it as a general psychopathological risk factor (Jacobi and Fittig, 2012; Machado et al., 2014).

Lastly, two psychological variables, perfectionism and drive for thinness, and one biological variable, T3, appeared to be specific correlates associated with the onset of an ED. The literature broadly supports the role of these variables. Indeed, a recent study has revealed that Free-T3 is a specific and sensitive correlate in distinguishing constitutional thinness and AN groups, showing significantly lower values in the latter with similar BMI between groups (Estour et al., 2017). Thus, although the low levels are thought to normalize with weight gain, evidence shows its relevance in AN patients and therefore, an early assessment in adolescents with a suspected ED is advisable.

The current study presents several limitations. Firstly, the cross-sectional case-control nature of the study does not allow inferring causality. However, Jacobi et al. (2004) suggested that using a case-control study is a good way to analyze correlates in a wide sample that can then be contrasted in a longitudinal study. Secondly, we only considered patients with a maximum of a 1-year course in order to reduce bias due to retrospective recall, although some of them had a history of a previous psychiatric disorders. Consequently, the involvement of other informants, such as parents, is important to contrast the information given by the adolescents. Thirdly, females with high socioeconomic status were predominant in this sample. Although it may be a limitation for the generalization of the results, high socioeconomic status is also frequent in EDs (Striegel-Moore and Bulik, 2007), and matching for parental socioeconomic status reduces differences in family experiences related to the availability of resources.

To summarize, this study proposes a complex model, which shows the importance of different correlates that are associated with the onset of an ED, although our findings require further research that can be contrasted in longitudinal studies and assessed in comparison with other control groups in order to confirm the specificity of the correlates. Most of the correlates found in this study are a replication of previously found risk factors in the literature, whereas the specificity and their relation have not been fully investigated. All of the participants have been assessed with reliable measures (blood test, clinical interview, and questionnaires). In our bio-psycho-familial model, eight correlates were specifically related to ED, therefore, the study confirms the importance of these three types of variables, which could be the target of future prevention and treatment interventions.

## Data Availability Statement

The original contributions presented in the study are included in the article/supplementary material, further inquiries can be directed to the corresponding authors.

## Ethics Statement

The studies involving human participants were reviewed and approved by approval was granted by the Ethic Committee of the Niño Jesús University Children’s Hospital (Ref Code. R-0009/10) and by the Autonomous University of Madrid Ethic Research Committee (UAM, CEI 25-673). Written informed consent to participate in this study was provided by the participants’ legal guardian/next of kin.

## Author Contributions

AS, EN, AM, JV-A, EM, and MG contributed to conception and design of the study. DA, SG-M, and AM organized the database. JMH performed the statistical analysis. AM wrote the manuscript. All authors contributed to manuscript revision, read, and approved the submitted version.

## Conflict of Interest

The authors declare that the research was conducted in the absence of any commercial or financial relationships that could be construed as a potential conflict of interest.

## Publisher’s Note

All claims expressed in this article are solely those of the authors and do not necessarily represent those of their affiliated organizations, or those of the publisher, the editors and the reviewers. Any product that may be evaluated in this article, or claim that may be made by its manufacturer, is not guaranteed or endorsed by the publisher.
